# Laboratory evidence on the vector competence of European field-captured *Culex theileri* for circulating West Nile virus lineages 1 and 2

**DOI:** 10.1186/s13071-025-06763-6

**Published:** 2025-04-05

**Authors:** Albert Burgas-Pau, Jaume Gardela, Carles Aranda, Marta Verdún, Raquel Rivas, Núria Pujol, Jordi Figuerola, Núria Busquets

**Affiliations:** 1 IRTA, Animal Health, Centre de Recerca en Sanitat Animal (CReSA), Campus de la Universitat Autònoma de Barcelona (UAB), 08193 Bellaterra, Catalonia Spain; 2 Unitat Mixta d’Investigació IRTA-UAB en Sanitat Animal, Centre de Recerca en Sanitat Animal (CReSA), Campus de la Universitat Autònoma de Barcelona (UAB), 08193 Bellaterra, Catalonia Spain; 3 Servei de Control de Mosquits del Consell Comarcal del Baix Llobregat, El Prat de Llobregat, Spain; 4 Department of Wetland Ecology, Estación Biológica Doñana, Seville, Spain; 5 CIBER Epidemiología y Salud Pública (CIBERESP), Madrid, Spain

**Keywords:** West Nile virus, *Culex theileri*, Vector competence, Arbovirus, Transmission

## Abstract

**Background:**

*Culex theileri* (Theobald, 1903) is distributed in Afrotropical, Paleartic, and Oriental regions. It is a mainly mammophilic floodwater mosquito that is involved in the transmission of West Nile virus (WNV, renamed as *Orthoflavivirus nilense* by the International Committee on Taxonomy of Viruses [ICTV]) in Africa. This virus is a mosquito-borne flavivirus that is kept in an enzootic cycle mainly between birds and mosquitoes of the *Culex* genus. Occasionally, it affects mammals including humans and equines causing encephalopathies. The main purpose of the present study was to evaluate the vector competence of a European field-captured *Cx. theileri* population for circulating WNV lineages (1 and 2).

**Methods:**

Field-collected *Cx. theileri* larvae from Sevilla province (Spain) were reared in the laboratory under summer environmental conditions. To assess the vector competence for WNV transmission, 10–12 day old *Cx. theileri* females were fed with blood doped with WNV lineages 1 and 2 (7 log_10_ TCID_50_/mL). Females were sacrificed at 14- and 21- days post exposure (dpe), and their head, body, and saliva were extracted to assess infection, dissemination, and transmission rates, as well as transmission efficiency.

**Results:**

A *Culex theileri* population was experimentally confirmed as a highly competent vector for WNV (both lineages 1 and 2). The virus successfully infected and disseminated within *Cx. theileri* mosquitoes, and infectious virus isolated from their saliva indicated their potential to transmit the virus. Transmission efficiency was 50% for lineage 1 (for both 14 and 21 dpe), while it was 24% and 37.5% for lineage 2, respectively. There was barely any effect of the midgut infection barrier for lineage 1 and a moderate effect for lineage 2. The main barrier which limited the virus infection within the mosquito was the midgut escape barrier.

**Conclusions:**

In the present study, the high transmission efficiency supports that *Cx. theileri* is competent to transmit WNV. However, vector density and feeding patterns of *Cx. theileri* mosquitoes must be considered when estimating their vectorial capacity for WNV in the field.

**Graphical Abstract:**

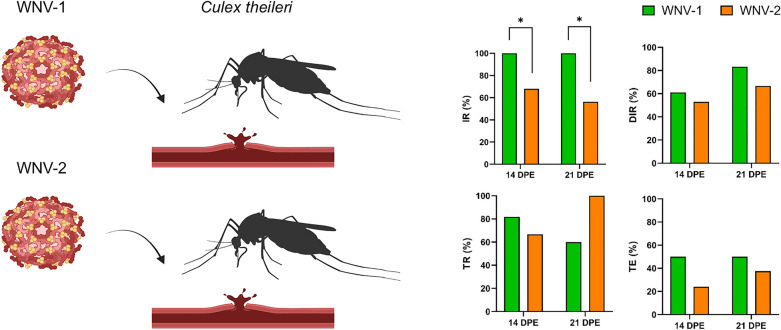

**Supplementary Information:**

The online version contains supplementary material available at 10.1186/s13071-025-06763-6.

## Background

*Culex theileri* (Theobald, 1903) is a floodwater mosquito distributed in south, east, and north Africa, as well as Palearctic, Middle Eastern, and east Oriental regions [1]. It is a polycyclic species that can be found in a broad range of elevations. The larvae occur in spring in places with stagnant water, and usually breed in fresh or slightly saline water. Several pathogens of medical and veterinarian importance have been detected from *Cx. theileri* mosquitoes, including Sindbis virus, West Nile virus (WNV), Rift Valley Fever virus [1, 2], *Dirofilaria immitis* [3], and *Plasmodium *spp. [4]. Vector competence of *Cx. theileri* for WNV lineage 2 (WNV-2) collected near Johannesburg was demonstrated by feeding on viraemic chicks, suggesting it as a potential vector of WNV in South Africa [5, 6]. However, to the best of our knowledge, no other information for other *Cx. theileri* populations is available for Europe [7] nor for any other continent.

West Nile virus (recently renamed as *Orthoflavivirus nilense* [8]) is a mosquito-borne arbovirus belonging to the *Orthoflavivirus* genus in the family *Flaviviridae* [9]. WNV has a wide geographical range and today it is found commonly in Africa, Europe, the Middle East, North America, and West Asia. WNV is transmitted mainly between mosquitoes of the *Culex* genus, that act as vectors, and birds as the principal reservoir hosts [10–12]. Humans and equids are considered dead-end hosts, which means that in infected individuals the virus is not able to reach enough viremia to be spread to mosquitoes. Luckily, most of WNV infections in humans are asymptomatic, although in elderly or immunocompromised individuals, as well as in some horses, neurological disorders can also result in encephalitis, meningitis, or even death [13, 14].

Although the vector competence of *Cx. theileri* has been proven for South African mosquitoes [5, 6], it is not generally assumed to play an important role in WNV transmission in the field owing to its ecological traits. *Culex theileri* is mainly a mammophilic mosquito that feeds occasionally on birds, behavior that has been described in different countries, such as Iran [15], Portugal [16], and Spain [17, 18]. This makes it difficult for *Cx. theileri* to enter into the transmission cycle of WNV, as the virus is maintained in a transmission cycle between birds and mosquitoes [19]. WNV field surveillance data in Europe further support the low implication of *Cx. theileri* in WNV circulation in Europe [20], with no detection of WNV-positive *Cx. theileri* despite extensive testing in Italy, Spain, and Türkiye [21, 22].

Several lineages of the WNV have been identified in Europe; the primary lineages that infect humans are 1 and 2. The co-circulation of WNV lineage 1 (WNV-1) and 2 (WNV-2), the recent expansion of WNV into more northern regions of Europe outside of its endemic regions, and the yearly recurrence of outbreaks makes WNV a public health concern in Europe [23]. At least 13 introductions of WNV-1 and WNV-2 have taken place into Europe, compared with only one introduction of WNV-1 in North America, resulting in the spread of the virus to a new continent [24]. In Spain, WNV was first detected in humans in 2004 [25], birds in 2007 [26], mosquitoes in 2006 [27], and again in both humans (two cases) and horses (36 affected herds) [28] in 2010. Since then, WNV-1 has been considered endemic in the southwestern region [29]. In 2017, WNV-2 was detected for the first time in Catalonia (northeastern Spain) in a northern goshawk [30] and has been able to persist in this region [31].

The current climate change situation can alter both the temporal dynamics of circulation of WNV and the abundance and distribution of its vectors. A higher spring temperature has been related with an earlier detection of WNV [32], as well as with an earlier start of a *Culex pipiens* season and increased season length in northern Italy [33, 34]. In southern Spain, a long-term study revealed the correlation between warm winters and springs and a higher WNV detection in host birds [35] and horses [36]. In 2020, the WNV epidemiological situation in Spain worsened, with 77 cases in humans, including seven deaths [37], evidencing the potential impact of WNV on public health. This situation was related to a high density of *Culex perexiguus* near the affected area, and retrospective analyses of collected mosquitoes found 33 pools of this species and 1 pool of *Cx. pipiens* infected with WNV [38]. Even *Cx. theileri* seemed not unrelated to 2020’s outbreak in Sevilla; it is present in high densities in the affected area during the spring season, that is, its peak abundance occurs during May, June, and July [39], before WNV peak season, which reaches its height in the northern hemisphere around July–September [40].

The main purpose of the present study was to evaluate the vector competence of a European field-collected *Cx. theileri* population for circulating WNV-1 and WNV-2, simulating summer conditions in Sevilla, to better estimate its potential role in WNV transmission.

## Methods

### Mosquito rearing and characterization

A *Culex theileri* population was obtained by rearing larvae collected in Puebla del Río (37°16′ N, 6°03′ W) and Coria del Río (37°17′ N, 6°03′ W) (Sevilla province) in June 2022. Larvae were reared in trays containing 750 mL of dechlorinated water supplemented with food fish (Tetra Goldfish, Melle, Germany), with each tray containing 200 larvae. Mosquitoes were maintained in the laboratory under controlled environmental conditions following the environmental conditions in the Sevilla province in summer, calculated on the basis of the mean conditions from July to September 2020 and 2021: 14:10 (L:D) photoperiod, 29–23 °C temperature ,and 70% relative humidity. These data were obtained from the EU’s Copernicus programme (https://www.copernicus.eu/).

The colony was started from field-collected larvae. When larvae emerged as adult individuals, all those that had progeny after blood feeding were characterized morphologically [41] and a subset of them (10 out of 66) were molecularly confirmed by polymerase chain reaction (PCR) and sequencing of a 710 bp fragment of the mitochondrial cytochrome c oxidase subunit I gene of the *Cx. theileri* species. Briefly, DNA from progenitors was extracted from legs and wings following the procedure described in Vogels et al. [42]. PCR was performed using LCO1490 and HCO2198 primers as previously described [43]. PCR reactions were carried out using Biotools DNA polymerase (Biotools, Madrid, Spain) in 25 µL reaction volumes: 2.5 µL buffer (10×), 0.25 µL MgCl_2_, 1.25 µL forward primer (10 µM), 1.25 µL reverse primer (10 µM), 0.4 µL Taq polymerase, 0.5 µL dNTPs mix (10 µM) (Biotools, Madrid, Spain), 18.35 µL nuclease-free water, and 0.5 µL template DNA. PCR thermal cycling conditions were as follows: 95 °C for 5 min; followed by 35 cycles of 95 °C for 1 min, 40 °C for 1 min, and 72 °C for 1 min 30 s; and 72 °C for 7 min. DNA of PCR products were quantified by Biodrop (Fisher Scientific, Pittsburgh, USA) and sequenced (Macrogen, Seoul, KR).

A previous study performed between 2001 and 2005 in Spain identified 50 insect-specific flavivirus positive pools of *Cx. theileri* [44]. Thus, a subset of the progenitors (10 out of 66) was screened for flaviviruses, and also for *Wolbachia*, to confirm the absence of these infections and avoid their possible interference with the results of vector competence assay. Briefly, viral RNA was extracted from progenitors’ bodies using the NucleoSpin RNA Virus kit (Macherey–Nagel, Düren, Germany). PCR for flavivirus detection was performed using cFD2 and MAMD primers targeting a 250 bp fragment of the *NS5* gene [45]. PCR reactions were performed using the Qiagen One Step RT-PCR Kit (Qiagen, Hilden, Germany) in 25 µL reaction volumes: 5 µL One Step buffer, 2.5 µL Q solution, 0.5 µL dNTPs, 0.25 µL ribonuclease inhibitor (20U/µL) (Applied Biosystems, Massachusetts, USA), 1 µL enzyme mix, 1.25 µL forward primer (10 µM), 1.25 µL reverse primer (10 µM), 11.25 µL nuclease-free water, and 2 µL template RNA. PCR thermal cycling conditions were as follows: 50 °C for 30 min and 95 °C for 15 min; followed by 40 cycles of 94 °C for 30 s, 55 °C for 30 s and 72 °C for 30 s; and 72 °C for 7 min. DNA was extracted from progenitors’ bodies using the DNeasy Blood & Tissue kit (Qiagen, Hilden, Germany) following the instructions of the manufacturer. PCR for *Wolbachia* detection was performed using wsp 183F and wsp 691R primers, as previously described [46]. PCR reactions were carried out using BIOTAQ DNA Polymerase (Bioline, London, UK) in 20 µL reaction volumes: 2.5 µL buffer (10×), 2 µL MgCl_2_, 1 µL forward primer (10 µM), 1 µL reverse primer (10 µM), 0.5 µL dNTPs (Bioline, London, UK), 0.2 µL Taq polymerase, 10.8 µL nuclease-free water, and 2 µL template DNA. PCR thermal cycling conditions were as follows: 94 °C for 3 min; followed by 35 cycles of 94 °C for 1 min, 55 °C for 1 min and 72 °C for 1 min; and 72 °C for 7 min.

### Viruses

WNV-1 and WNV-2 were used to assess the vector competence of *Cx. theileri* mosquitoes. WNV-1 [SPA-E-2020-01, kindly provided by Miguel Ángel Jiménez-Clavero, INIA-CISA, and first isolated at *Laboratorio Central de Veterinaria de Algete* (Madrid)] was isolated from the brain of a cinereous vulture (*Aegypius monachus*), from Cádiz in 2020, on Vero 76 CRL-1587 cells (ATCC, Virginia, USA) and propagated at the *Institut de Recerca i Tecnologia Agroalimentàries—Centre de Recerca en Sanitat Animal* (IRTA-CReSA, Barcelona) once on Vero CCL-81 cells (ATCC, Virginia, USA). WNV-2 [AC924, first isolated at *Institut de Recerca i Tecnologia Agroalimentàries—Centre de Recerca en Sanitat Animal* (IRTA-CReSA, Barcelona)] was isolated from the brain of a northern goshawk (*Accipiter gentilis*) from Tarragona in 2020 and was passaged one time on Vero CCL-81 cells (ATCC, Virginia, USA).

### Vector competence assay

*Culex theileri* females (F3) were artificially fed with chicken blood doped with WNV from a frozen viral stock when 10–12 days old. The final concentration for both WNV-1 and WNV-2 was 7 log_10_ TCID_50_/mL. This titer was used as suggested by Vogels et al. to compare the outcomes of different vector competence studies for WNV [7]. Before virus exposure, sucrose starvation was performed for 4 days, mosquito females were allowed to drink water for the first 72 h of sucrose starvation, and no food or water was provided the last 24 h before blood feeding. The viral exposure was performed using a Hemotek feeding system (Discovery Workshop, Accrington, UK) set at 37.5 °C for 1 h. Blood-engorged females were anesthetized with CO_2_, separated into groups of 10 in cardboard cages (Watkins & Doncaster, Leominster, UK), and kept under the above-mentioned rearing conditions. Throughout the experiment, mosquitoes were maintained with 10% sucrose solution ad libitum. Three females of each group were sacrificed and conserved as 0 dpe samples to ensure the presence of the virus by RT-qPCR as described below.

A similar number of females exposed to WNV-1 or WNV-2 were sacrificed 14- and 21-dpe. Legs and wings were removed and saliva was extracted using a pipette tip with 7 µL of fetal bovine serum (FBS) (Gibco Life Technologies, Massachusetts, USA). The proboscis was introduced into the tip for 30 min and saliva was stored in 193 µL of Dulbecco’s modified Eagle’s medium (DMEM) (Gibco, Massachusetts, USA) supplemented with 1× antibiotic–antimycotic (Gibco Life Technologies, Massachusetts, USA; 100×, containing 10,000 units/mL of penicillin, 10,000 µg/mL of streptomycin, and 25 µg/mL of amphotericin B). Heads and bodies were collected in 500 µL of DMEM (Gibco, Massachusetts, USA) with 1× antibiotic–antimycotic (Gibco Life Technologies, Massachusetts, USA; 100×) and glass beads (LabComercial, Barcelona, Spain), homogenized at 30 Hz for 1 min using TissueLyser II (Qiagen GmbH, Hilden, Germany) and maintained at –80 °C (as with saliva samples) until virus isolation. To assess the vector competence, several indexes were assessed: the infection rate (IR) as the proportion of mosquitoes with an infected body among all the blood-feed mosquitoes; the disseminated infection rate (DIR) as the proportion of mosquitoes with an infected head among the ones with an infected body; the transmission rate (TR) as the proportion of mosquitoes with infectious saliva among the ones with an infected head; and the transmission efficiency (TE) as the proportion of mosquitoes with infectious saliva among all the mosquitoes exposed to the virus analyzed.

The experimental infections were carried out at the *Institut de Recerca i Tecnologia Agroalimentàries—Centre de Recerca en Sanitat Animal* (IRTA-CReSA) BLS3 facilities.

### West Nile virus detection

Virus detection in head and body samples was performed by inoculation of 10-fold and 100-fold dilutions on 96 well plates with Vero CCL-81 cells (ATCC, Virginia, USA). Inoculated Vero cells were maintained using DMEM (Gibco, Massachusetts, USA), which was supplemented with 1% l-glutamine (Gibco, Massachusetts, USA), 2% FBS (Gibco Life Technologies, Massachusetts, USA), and 1× antibiotic–antimycotic (Gibco Life Technologies, Massachusetts, USA; 100 ×) for 7 days at 37 °C and 5% CO_2_. Then, the cytopathic effect (CPE) was visually evaluated.

Virus detection in saliva samples was performed by inoculation of 35 µL of each saliva sample on six-well plates with Vero CCL-81 cells (ATCC, Virginia, USA). Inoculated cells were maintained using DMEM (Gibco, Massachusetts, USA) supplemented with 2% FBS (Gibco Life Technologies, Massachusetts, USA), 1% l-glutamine (Gibco, Massachusetts, USA), 1% sodium pyruvate (Sigma Aldrich, Missouri, USA), 2.5% sodium bicarbonate (Sigma Aldrich, Missouri, USA), 1% lactalbumin hydrolysate (Sigma Aldrich, Missouri, USA), 1% noble agar (BD, MD, USA), and 1× antibiotic–antimycotic (Gibco Life Technologies, Massachusetts, USA; 100×) for 7 days at 37 °C and 5% CO_2_. Then, a solution of paraformaldehyde (2%) and crystal violet (0.1%) was added overnight to fix the cell monolayers. The following day the agar plugs were removed to observe the CPE. Viral titers from saliva samples were expressed as plaque forming units per volume (PFUs/mL).

Real-time quantitative reverse transcription PCR (RT-qPCR) was performed to detect WNV viral RNA in body and head samples. Briefly, viral RNA was extracted from samples using the NucleoSpin RNA Virus kit (Macherey–Nagel, Düren, Germany) following the manufacturer’s instructions. RT-qPCR was performed using FLI-WNF5-F and FLI-WNF6-R primers, and the FLI-WNF probe targeting the non-structural NS2A region of WNV, as previously described [47]. RT-PCR reactions were carried out using AgPath-ID One-Step RT-PCR reagents (Applied Biosystems, Massachusetts, USA) in 20 µL reaction volumes: 10 µL buffer (2×), 0.8 µL enzyme mix (25×), 2 µL forward primer (10 µM), 2 µL reverse primer (10 µM), 0.25 µL probe (10 µM), 1.37 µL IPC—Ribosomal RNA Control Reagents (Applied Biosystems, Massachusetts, USA), and 3.58 µL template RNA. PCR thermal cycling conditions were as follows: 45 °C for 10 min and 95 °C for 10 min; followed by 40 cycles of 95 °C for 15 s and 60 °C for 1 min. To determine the virus concentration in each sample, a calibration curve was created using repeated tenfold dilutions of the RNA standard (oligonucleotide purchased from Eurogentec and Integrated DNA Technologies, Seraing, BEL) with a known concentration. The number of copies of virus in each sample was then determined.

### Statistical analysis

The effects of virus lineage and days post exposure (predictors) on infection, dissemination, and transmission rates, and transmission efficiencies (response variable) were tested using generalized linear models (GLM) with binomial distributed errors and the logit link function.

Saliva viral titers could not be normalized using common transformations, as evaluated by Shapiro–Wilk test, and mean saliva viral titers were compared between the two lineages at different time-points using the Wilcoxon rank-sum test.

GLMs with binomial distributed errors and the logit link function were fitted to test the relationship between body and head viral copies (predictors) and the positivity of saliva samples tested by CPE in Vero cells (response variable). Linear models (LMs) were used to describe the association between the number of viral copies of the body and head (predictors) and the titers of saliva samples (response variable).

All statistical analyses were carried out using R statistical software (http://cran.r-project.org/).

## Results

### *Culex theileri* mortality rate

In total, 46 and 60 engorged females were recovered after exposure to blood doped with WNV-1 and WNV-2, respectively. The presence of the virus was confirmed by RT-qPCR in the females of each group sacrificed at 0 dpe.

Mortality rates of 30.23% (13/43) and 28.07% (16/57) were observed throughout the assay in the groups exposed to WNV-1 and WNV-2, respectively.

### Estimation of the vector competence of *Cx. theileri* for West Nile virus lineages 1 and 2 after oral exposure

**Fig. 1:**
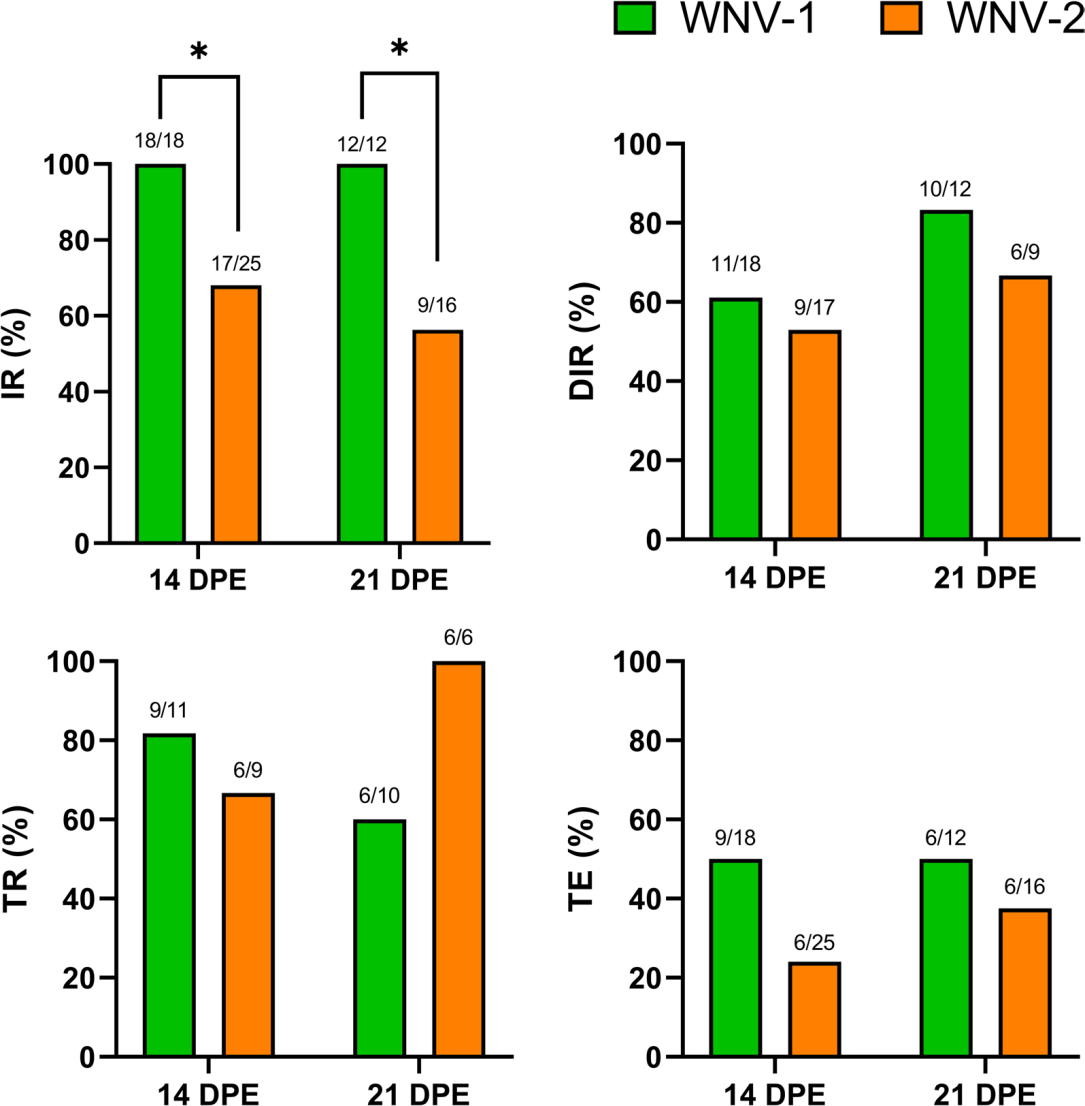
Infection, dissemination, and transmission rates, as well as transmission efficiency of a field-collected *Cx. theileri* population exposed to WNV lineages 1 (green) and 2 (orange bars). * Denotes statistically significant differences between lineages. IR, infection rate; DIR, dissemination rate; TR, transmission rate; TE, transmission efficiency

According to the vector competence rates assessed in the present study, European field-captured *Cx. theileri* was able to become infected, disseminated with, and transmit both WNV-1 and WNV-2 after an oral exposure of blood doped with an infectious viral dose of 7 log_10_ TCID_50_/mL (Fig. 1). WNV-1 was able to infect all of the mosquitoes at 14 (18/18) and 21 (12/12) dpe. In contrast, for WNV-2, the success in infecting mosquitoes was lower at both timepoints [68% (17/25) at 14 dpe and 56.25% (9/16) at 21 dpe]. The infection rate for mosquitoes exposed to WNV-1 was significantly higher (GLM, *Z* = −2.890, *df* = 1, *P* = 0.004), while days post-exposure did not have a significant effect (GLM, *Z* = −0.793, *df* = 1, *P* = 0.428). Moreover, the virus successfully disseminated throughout the hemolymph and could be detected in mosquito heads for both lineages. WNV-1 was able to disseminate within 11 out of 18 mosquitoes (61.11%) at 14 dpe, and 10 out of 12 (83.33%) at 21 dpe; and WNV-2 was disseminated in 9 out of 17 mosquitoes (52.94%) and in 6 out of 9 (66.67%) at 14 dpe and 21 dpe, respectively. The differences in disseminated infection rates between lineages (GLM, *Z* = −0.406, *df* = 1, *P* = 0.685) and days post-exposure (GLM, *Z* = 0.634, *df* = 1, *P* = 0.526) were not statistically significant. Finally, WNV-1 was detected in saliva in 9 out of 11 disseminated mosquitoes (81.82%) at 14 dpe, and 6 out of 10 (83.33%) at 21 dpe. For WNV-2, saliva tested positive in 6 out of 9 (66.67%) disseminated mosquitoes at 14 dpe, and 6 out of 6 (100%) at 21 dpe. The differences in transmission rates between lineages (GLM, *Z* = 0.220, *df* = 1, *P* = 0.826) and days post-exposure (GLM, *Z* = 0, *df* = 1, *P* = 1) were not statistically significant.

Regarding the main vector competence barriers (Table 1), the present results showed that the midgut infection barrier had a null effect, as it did not avoid the infection of midgut epithelial cells for WNV-1 and had a minor effect for WNV-2. Besides, the midgut escape barrier had a minor effect on avoiding the dissemination of WNV-1 and had a moderate effect on WNV-2 dissemination. In addition, transmission rates showed that salivary gland barriers had a low effect on avoiding the transmission of both WNV-1 and WNV-2.

**Table 1:** Infection, dissemination, and transmission rates, as well as transmission efficiency in *Cx. theileri* after oral exposure to WNV lineages 1 and 2

Lineage	IR (%)	DIR (%)	TR (%)	TE (%)
	MIB	MEB	SGB	
WNV-1	30/30 (100)	21/30 (70)	15/21 (71.43)	15/30 (50)
	Null	+	+	
WNV-2	26/41 (63.41)	15/26 (57.69)	12/15 (80)	12/41 (29.27)
	+	++	+	

Number positive for WNV/number tested (prevalence), and relative rating of the importance of the barrier

Rating of the relative importance of the barrier: null, virus crosses this barrier in > 80% of females; +, minor, virus crosses this barrier in 60–80% of females; ++, moderate, virus crosses this barrier in 40–60% of females [81]

IR, infection rate; DIR, disseminated infection rate; TR, transmission rate; TE, transmission efficiency; MIB, midgut infection barrier; MEB, midgut escape barrier; SGB, salivary gland barriers

Regarding the transmission efficiency (Fig. 1) of WNV-1, 50% of engorged females were able to transmit the virus at both 14 dpe and 21 dpe. For WNV-2, 24% of engorged females could transmit the virus at 14 dpe and 37.5% at 21 dpe. These results reflect a high overall vector competence of the *Cx. theileri* population for both WNV-1 and WNV-2. The differences in transmission efficiency between lineages (GLM, *Z* = −1.759, *df* = 1, *P* = 0.078) and days post-exposure (GLM, *Z* = 0.673, *df* = 1, *P* = 0.501) were not statistically significant.

### Evaluation of the viral titers of saliva samples

**Fig. 2:**
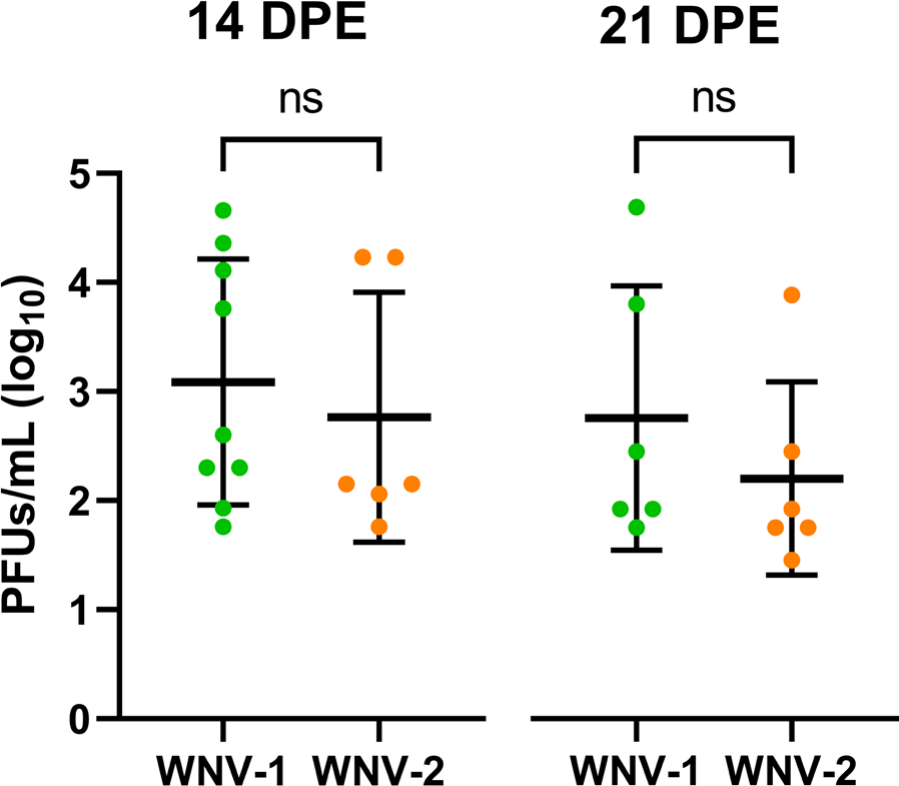
WNV loads in saliva (plaque forming units [PFU/mL]) of infected *Cx. theileri* females exposed to WNV lineages 1 and 2. DPE, days post-exposure; ns, non-significant

The females exposed to WNV-1 and WNV-2 showed similar viral titers both at 14 dpe (Wilcoxon rank sum test, *W* = 33.5, *P* = 0.478) and 21 dpe (*W* = 24.5, *P* = 0.329) (Fig. 2). The mean titer values at 14 dpe were 3.09 log_10_ PFU/mL and 2.76 log_10_ PFU/mL for WNV-1 and WNV-2, respectively, and at 21 dpe were 2.77 log_10_ PFU/mL and 2.21 log_10_ PFU/mL for WNV-1 and WNV-2, respectively.

### Relationship between body and head viral copies and transmission ability

The number of viral copies in the body (log_10_ transformed) (Additional file 1, Supplementary Table S1) was positively correlated with the presence of viable viral particles in saliva (Table 2, model 1). Increasing one unit of log_10_ viral copies in bodies was associated with 8.53 more odds of finding infectious virus in saliva titration. No differences were found between the lineages.

**Table 2:** Logistic regression models performed to assess the relationship between the detection of viable viral particles in saliva and the viral copies in bodies and heads and lineage of the virus

Model	Dependent variable (reference)	Factors (reference)	Estimate	Std Error	*Z*	OR	95% CI	*P*
1	Saliva (negative saliva)	Log_10_ viral copies in body	2.14	0.76	2.81	8.53	1.98–23.59	0.005*
		Lineage (WNV-2)	−0.81	0.57	−1.42	0.45	0.14–1.33	0.156
2	Saliva (negative saliva)	Log_10_ viral copies in head	4.01	1.16	3.44	54.92	8.06–738.39	0.001*
		Lineage (WNV-2)	−0.6	0.7	−0.85	0.55	0.13–2.21	0.398

Std Error, standard error; *Z*, *Z*-value; OR, odds ratio; 95% CI, 95% confidence interval; *P*, *P*-value

^*^Statistically significant

The relationship between log_10_ viral copies in the head (Additional file 1, Supplementary Table S1) and viable viral particle detection in saliva was also positive, with no differences between lineages (Table 2, model 2). Increasing one unit of log_10_ viral copies in the head was associated with 54.92 more odds of finding infectious virus in the saliva titration.

Finally, neither log_10_ viral copies in the body (LM, *t* = 1.996, *df* = 24, *P* = 0.057) or log_10_ viral copies in the head (LM, *t* = 0.637, *df* = 24, *P* = 0.530) were correlated with the titers of saliva samples (Additional file 1, Supplementary Table S1), expressed in log_10_ PFUs/mL. This was independent of the lineage (LM, *t* = −1.454, *df* = 24, *P* = 0.159, for bodies; LM, *t* = −1.248, *df* = 24, *P* = 0.224 for heads).

## Discussion

To the best of our knowledge, we present the first estimates of WNV vector competence in European field-captured populations of *Cx. theileri* [7]. According to our results, *Cx. theileri* showed a potential high vector competence for both lineages at the two assessed timepoints (14 and 21 dpe). This result can be explained by the minimal or even negligible impact of the different barriers the virus must overcome to infect different mosquito tissues. Although WNV-1 had a higher IR, no differences were found on DIR, TR, TE, nor on the viral titers of positive saliva samples between lineages. The lack of significance in the comparison of ratios between WNV-1 and WNV-2 may be likely due to the small sample sizes, since most statistical tests suffer from this problem.

Higher viral copies in the body or head were associated with an increased likelihood of detecting infectious viral particles in the saliva, but were not correlated with the titers of saliva samples. The virus titers that we obtained in saliva of *Cx. theileri* are in line with those found in other *Culex* species and were sufficient to infect a bird [48, 49], which might suggest *Cx. theileri* may infect susceptible birds, if they feed on them.

There is little evidence regarding the vector competence of *Cx. theileri* for WNV. Transmission studies performed in the 1960s on South-African populations of *Cx. theileri* exposed to WNV- 2 provide the foundation of our current knowledge [5, 6]. In the first study, the ability of mosquitoes to transmit the virus was assessed by exposing hen chicks to the bites of a variable number of infected females and detecting antibodies in the chick at 20- and 22-dpe. However, this approach leads to uncertainty in estimating transmission efficiency, as each chick was exposed to between one and six mosquitoes. Thus, reported transmission efficiency ranged between 3.125% and 18.75%. The second study, focused on the impact of viral dose, showed a 25% transmission efficiency at the highest viral dose tested, with no transmission observed at lower doses. A further limitation of both studies was the vague definition of the viral dose used for the experiments, which was only expressed in terms of logarithms without a clear indication of the viral titration method used. Despite these limitations, both studies succeed in reflecting the WNV transmission process occurring in nature. Our results are consistent with their findings, as we observed transmission efficiencies of 24% at 14 dpe and 37.5% at 21 dpe for WNV-2, and even higher for WNV-1, confirming that *Cx. theileri* is highly competent to transmit WNV.

The lack of studies performed on *Cx. theileri* with WNV-1 should be noted. Our results demonstrate that WNV-1 is significantly more efficient than WNV-2 in infecting *Cx. theileri* at both evaluated timepoints (while no significant differences were found in dissemination or transmission). This contrasts with results from previous studies on vector competence in different mosquito species with both lineages. For instance, no significant differences in terms of infection between WNV lineages were reported for *Aedes punctor* [50] or *Aedes vexans* [51], while *Aedes albopictus* and *Cx. pipiens* showed higher infection rates for WNV-2 [52].

When comparing our results on vector competence for *Cx. theileri* with studies on other *Culex* species, vector competence for WNV after oral exposure to infectious blood varies across European mosquito populations and species. For instance, studies conducted in similar environmental conditions as our experiment, but in *Cx. pipiens* populations from the Netherlands [53] and Germany [54], showed transmission efficiencies of 33% and 6.7–52.9%, respectively. However, populations of the same species exposed to similar environmental conditions from Italy and the Netherlands showed lower transmission efficiencies, ranging from 2% to 16% [55], while Finnish and Belgian populations showed transmission efficiencies ranging from 7% to 17% [56] and 4.3% [57], respectively. Taken together, these results point out that the vector competence of the *Culex* mosquito species for WNV is strongly dependent on the mosquito population and species. The effect of mosquito population in determining vector competence was clearly highlighted in a study conducted in northeastern France [58]. A similar pattern was observed in other European *Culex* mosquitoes under similar environmental conditions. For instance, a Belgian *Culex modestus* population was unable to transmit WNV [57], while French populations showed high vector competence for WNV, with transmission rates of 40% [59] and 54.5% [60].

Regarding the effect of the temperature in vector competence studies of the *Culex* mosquito species, studies performed on *Culex torrentium* from Germany [61] and Finland [56] were unable to transmit WNV at 18 °C and 21 °C. However, when exposed to the higher temperatures of 24 °C and 27 °C, they displayed significantly greater vector competence, with transmission efficiencies ranging from 2.9% to 33%. A similar temperature-pattern has been observed in several studies on *Cx. pipiens*, which show that the form pipiens was unable to transmit WNV at 18 °C but demonstrated WNV transmission at 23 °C and 28 °C [55, 62]. Interestingly, while the hybrid form followed the same trend, *Cx. pipiens* form molestus was able of transmitting WNV at the three tested temperatures with no significant differences [62]. All in all, these studies highlight the role of temperature as a key factor influencing vector competence in *Culex* mosquitoes. This pattern is also reflected in our results, where *Cx. theileri* displayed a high transmission efficiency, ranging from 24% to 50% under high temperatures (29 °C during the day and 23 °C at night). It should be noted that the same assay performed at lower temperatures would probably lead to lower transmission efficiencies. These findings emphasize the importance of considering environmental temperature in arbovirus vector competence studies [63] and suggest that warmer climates may enhance the role of *Cx. theileri* in WNV transmission, which in general may explain the association between WNV human case incidence and temperature, as reported in different studies in Europe [64].

It is known that the viral dose in the blood used for mosquito feeding is also a key factor influencing vector competence, as has been demonstrated in different studies on *Culex quinquefasciatus* [65], *Cx. pipiens*, and *Ae. albopictus* for WNV [52]; and *Ae. albopictus* and *Aedes aegypti* for Chikungunya virus (CHIKV) [66]. Likewise, our results showed a relationship between the number of viral copies in mosquito tissues and transmission ability, that is, the higher the number of viral copies within mosquito tissues, the greater the likelihood of detecting infectious virus particles in saliva. Thus, this correlation indicates that a high number of viral copies in mosquito tissues significantly increases the likelihood of an exposed female to transmit WNV. Understanding this relationship could be useful for developing strategies to limit the transmission of WNV, since the alteration of the number of viral copies within mosquitoes could disrupt the ability of a mosquito to transmit the virus. Some strategies are being adopted in this way for other arboviruses, for example, the use of bacteria such as *Wolbachia* has been shown to reduce viral copies of CHIKV, dengue virus, and yellow fever virus in *Ae. aegypti* [67, 68], and the use of double-strand RNA has been successful in blocking Zika virus infection in *Ae. aegypti* [69].

Considering all the evidence discussed, *Cx. theileri* shows an overall vector competence for WNV that is comparable to, and in some cases exceeds, that of other *Culex* species (considered the main vectors of WNV in Europe). Nevertheless, it is important to emphasize that vector competence is not enough to define the role of a mosquito species as a vector. The concept of vectorial capacity provides a more comprehensive framework, as it incorporates not only vector competence but also other factors such as vector density, biting rate, extrinsic incubation period of the pathogen, and daily survival probability [70]. Thus, despite its high vector competence for WNV, *Cx. theileri* is not currently considered a key player in WNV circulation and transmission owing to its ecological features, especially its feeding preferences, which are mainly mammophilic. However, field-captured WNV-positive *Cx. theileri* mosquitoes have been collected in South Africa [2, 71] and Iran [72], indicating its capacity to acquire the virus under natural conditions. This fact, added to the high competence for WNV, points out the need to consider the potential role of *Cx. theileri* as a vector in a context of elevated densities and high WNV circulation, a high overlap phenomenon that, despite not existing currently, could be promoted by the effects of climate change. Our results suggest the need to reevaluate the role of *Cx. theileri* on WNV transmission under conditions of high WNV circulation, e.g., under epidemic conditions. Indeed, several studies reported the coexistence of high densities of *Cx. theileri* with other *Culex* vectors of WNV, such as *Cx. perexiguus* and *Cx. pipiens* in Spain [18, 39] and *Cx. pipiens* in Türkiye [73], although their population density peaks do not fully overlap during the WNV transmission season.

WNV is the most widespread emerging arbovirus in the world [74], threatening human and animal health. Besides the existence of vaccines available to protect horses [75, 76], more prevention and treatment tools are needed to face virus emergence and reduce potential WNV epizootics and epidemics. Host–vector–host experimental models can be an excellent method to fill the gaps, as they replicate the natural barriers WNV encounters in nature, both in the vector and in the host. In addition, these models are useful to assess pathogenesis (such as the level of viremia or the immune response) or vaccine efficacy at the individual level. For instance, similar models have been performed to study Rift Valley fever virus with lambs and *Ae. aegypti* [77, 78]; dengue virus with mice and *Ae. aegypti* [79, 80]; and WNV with chicken, mice, *Culex tarsalis*, *Cx. pipiens*, *Aedes japonicus*, and *Aedes triseriatus* [48]. Since *Cx. theileri* was easy to rear and owing to its high vector competence for WNV, it may be a good model species to use in experimental models.

## Conclusions

The present study supports for the first time that a European field-captured *Cx. theileri* population is highly competent to transmit WNV-1 and WNV-2 under laboratory conditions, even though there is no evidence of its involvement in the current circulation of the virus in Europe. The insights around the competence of this mosquito species can contribute to its use in developing mosquito transmission models for WNV preventive measures. Further research is needed to elucidate the potential role of *Cx. theileri* in WNV circulation and transmission in the context of climate change and landscape use.

## Supplementary Information

Additional file 1: Table S1. Number of viral copies in the body and the head (log_10_ transformed), PFUs/ml in saliva (log_10_ transformed), positivity of the saliva, and lineage of WNV exposed for each sample.

## Data Availability

No datasets were generated or analyzed during the current study.

## Abbreviations

CHIKV: Chikungunya virus
CPE: Cytopathic effect
DIR: Disseminated infection rate
DMEM: Dulbecco’s modified Eagle’s medium
FBS: Fetal bovine serum
GLM: Generalized linear model
ICTV: International Committee on Taxonomy of Viruses
IR: Infection rate
L:D: Light:dark
MEB: Midgut escape barrier
MIB: Midgut infection barrier
PCR: Polymerase chain reaction
PFU: Plaque forming unit
RT-qPCR: Real-time quantitative reverse transcription PCR
SGB: Salivary gland barriers
TCID_50_: 50% Tissue culture infectious dose
TE: Transmission efficiency
TR: Transmission rate
WNV: West Nile virus
